# High Performance All Nonmetal SiN_x_ Resistive Random Access Memory with Strong Process Dependence

**DOI:** 10.1038/s41598-020-59838-y

**Published:** 2020-02-18

**Authors:** Te Jui Yen, Albert Chin, Vladimir Gritsenko

**Affiliations:** 1 Department of Electronics Engineering, National Chiao Tung University, Hsinchu, 300 Taiwan; 2 Rzhanov Institute of Semiconductor Physics. Siberian Branch, Russian Academy of Sciences, Novosibirsk, Russia; 3 Novosibirsk State University, Pirogova street, 2, Novosobirsk, 630090 Russia; 4 Novosibirsk State Technical University, K. Marx Ave., 20, Novosibirsk, 630073 Russia

**Keywords:** Electrical and electronic engineering, Electronic devices

## Abstract

All-nonmetal resistive random access memory (RRAM) with a N^+^–Si/SiN_x_/P^+^–Si structure was investigated in this study. The device performance of SiN_x_ developed using physical vapor deposition (PVD) was significantly better than that of a device fabricated using plasma-enhanced chemical vapor deposition (PECVD). The SiN_x_ RRAM device developed using PVD has a large resistance window that is larger than 10^4^ and exhibits good endurance to 10^5^ cycles under switching pulses of 1 μs and a retention time of 10^4^ s at 85 °C. Moreover, the SiN_x_ RRAM device developed using PVD had tighter device-to-device distribution of set and reset voltages than those developed using PECVD. Such tight distribution is crucial to realise a large-size cross-point array and integrate with complementary metal-oxide-semiconductor technology to realise electronic neurons. The high performance of the SiN_x_ RRAM device developed using PVD is attributed to the abundant defects in the PVD dielectric that was supported by the analysed conduction mechanisms obtained from the measured current–voltage characteristics.

## Introduction

Resistive random access memory (RRAM)^1–26^ has attracted considerable attention over the past two decades due to its simple structure, nonvolatility, high scalability, rapid switching speed, and relatively low operating power. These advantages make RRAM devices suitable for use in future artificial intelligence and neuromorphic computing applications^1–4^. A variety of binary composite materials, such as HfO_x_, TaO_x_, TiO_x_, SiO_x_, and GeO_x_, that exhibit different device properties have been used as the switching layer. Although various conduction mechanisms for RRAMs have been proposed, the carrier transport behaviour of RRAMs still has not been completely confirmed. To prevent the metal ions from contributing to transport behaviour, we pioneered nonmetal GeO_x_ dielectric RRAM devices^6–10^ and all-nonmetal N^+^–Si/SiO_x_/P^+^–Si RRAM devices^11^. In this study, we investigated the all-nonmetal SiN_x_ RRAM devices in which the bond enthalpy of SiN is lower than that of SiO^27^. The SiN_x_ material has been widely used in the semiconductor industry for various applications, such as the passivation layer of an integrated circuit and the charge-trapping layer of a flash memory. This material can be easily integrated into complementary metal-oxide-semiconductor (CMOS) technology. Moreover, the RRAM device performance strongly depends on the SiN_x_ formation process. A high-performance RRAM device with a large memory window, good endurance, long retention time, and tight device-to-device distribution of the set–reset voltages *V*_*set*_–*V*_*reset*_ is only achievable when the SiN_x_ layers are formed using low-temperature physical vapor deposition (PVD) rather than the standard plasma-enhanced chemical vapor deposition (PECVD). The high-performance SiN_x_ RRAM device developed using PVD (PVD-SiN_x_ RRAM device) is linked to the high number of defects and high amount of defect-related current conduction in the SiN_x_ dielectric.

## Results

**Figure 1:**
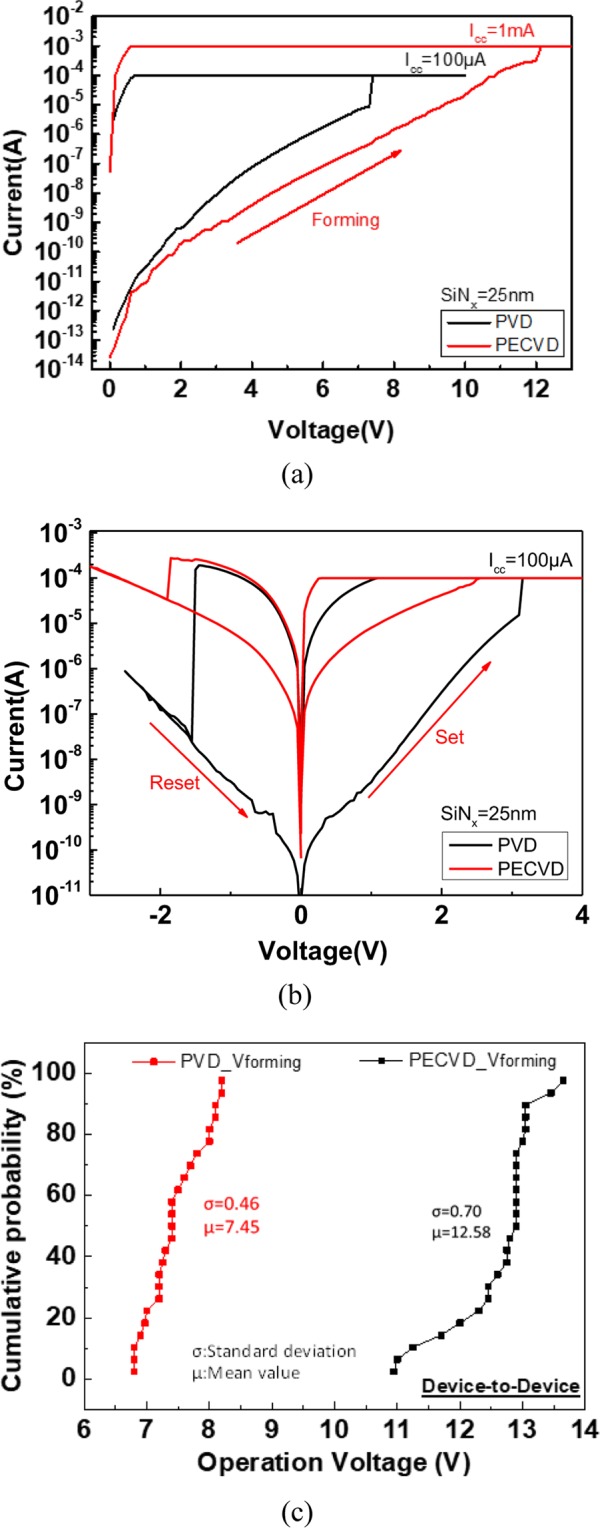
*I-V* characteristics of PVD and PECVD SiN_x_ RRAM devices of (**a**) forming process, (**b**) set-reset processes, and (**c**) forming voltage distributions.

Figure 1(a) displays the forming process of the PVD-SiN_x_ RRAM device and the SiN_x_ RRAM device developed using PECVD (PECVD-SiN_x_ RRAM device). While the forming step of fabricating the PECVD-SiN_x_ RRAM device, the *I*_*cc*_ value was increased from 100 μA with a 100-μA step until the resistance state could be switched to the low-resistance state (LRS) at an *I*_*cc*_ value of 1 mA. The PVD-SiN_x_ RRAM device exhibited lower *I*_*cc*_ and lower forming voltage than those of the PECVD-SiN_x_ RRAM device. These results reflect that the initial PVD-SiN_x_ RRAM device has more defects to form a conduction path than the PECVD-SiN_x_ RRAM device. Figure 1(b) depicts the typical bipolar switching characteristics of both PVD-SiN_x_ and PECVD-SiN_x_ RRAM devices. The PVD-SiN_x_ RRAM device exhibits a significantly lower high-resistance state (HRS) current and a larger resistance window than those of the PECVD-SiN_x_ RRAM device. The much higher HRS current in the PECVD-SiN_x_ RRAM device may be related to the higher *I*_*cc*_ and bias voltage values needed during the forming process, where such high power may damage the SiN_x_ and create unrecoverable defects. The asymmetric LRS *I-V* curve of the PECVD device may be due to the large forming voltage and power to generate excessive defects, which may be partially annihilated by injected electrons during negative voltage swept. Figure 1(c) shows the forming voltage distributions of PVD-SiN_x_ and PECVD-SiN_x_ devices, which are ranged from 6.8 to 8.2 V and from 11.0 to 13.7 V, respectively.

**Figure 2:**
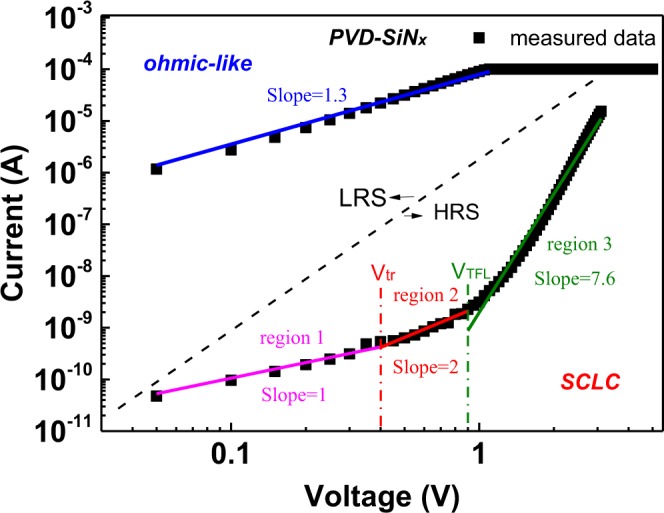
*I-V* characteristics of PVD-SiN_x_ RRAM device were analyzed by the space-charge limited conduction (SCLC) in HRS and ohmic-like behavior conduction in LRS.

**Figure 3:**
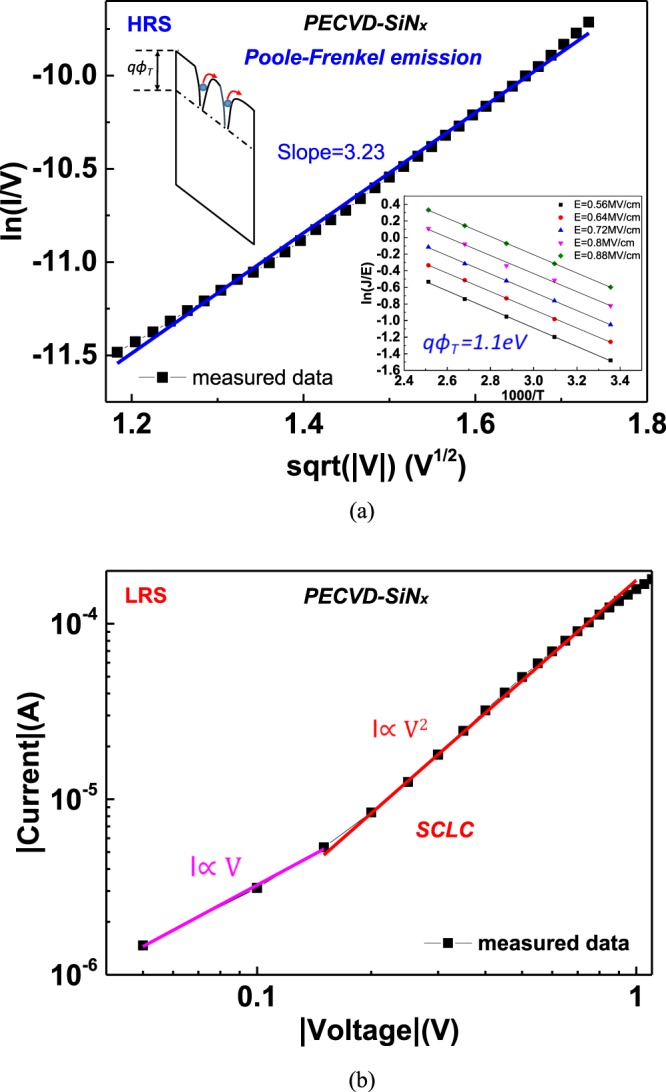
*I-V* curves of PECVD-SiN_x_ RRAM were analyzed with (**a**) Poole-Frenkel (P-F) emission in HRS and (**b**) space charge limited conduction in LRS. The inset figure in (**a**) is Arrhenius plot of the PF emission.

To further understand the carrier conduction mechanism, we analysed the measured data by using various transport mechanisms. Because there is no metal inside the dielectric to form the metallic filament, the conduction mechanism of nonmetal RRAM device is attributed to defect-related hopping conduction^10,12,13^. Figures 2 and 3 display the analysed conduction mechanisms for PVD-SiN_x_ and PECVD-SiN_x_ RRAM devices, respectively. The very low HRS current in the PVD-SiN_x_ RRAM devices, as presented in Fig. 2, is attributed to the space-charge limited conduction (SCLC) mechanism^14–16,28^. The SCLC regions are divided into three regions by using the transition voltage *V*_*tr*_ and trap-filled limited voltage *V*_*TFL*_. For the low-bias voltage region that is less than *V*_*tr*_, the current is proportional to voltage and follows Ohm’s law, with an extremely high resistance of approximately 10 GΩ. The current density–voltage (*J–V*) relationship obtained when the applied voltage is larger than *V*_*tr*_ but lower than *V*_*TFL*_ (region 2) can be expressed as follows^17,29^:

1 $${\rm{J}}=\frac{9\mu \varepsilon \theta }{8{d}^{3}}{V}^{2},\theta =\frac{{J}_{free}}{{J}_{trap}+{J}_{free}},$$

where *J*, *μ*, *ε*, *d*, and *θ* represent the current density, electron mobility, static dielectric constant, dielectric layer thickness, and ratio of the free current density to the trapped and free current densities, respectively. In this region, shallow traps are gradually filled by increasing the electric field, and the injected free carrier current density *J*_*free*_ significantly contributes to the total current density. All the traps at voltages larger than *V*_*TFL*_ are filled by injected carriers. A massive number of free carriers contribute to current conduction under a high electric field, thus leading to a steep current–voltage (*I–V*) slope (region 3) For the PVD device, the currents of HRS *I-V* curves increase with increasing temperature^18^. Conversely, the LRS, which exhibited ohmic-like behavior, can be analysed with a slope of 1.3^9,13^.

For the HRS current of the PECVD-SiN_x_ devices presented in Fig. 3(a), the current conduction resembles Poole–Frenkel (P–F) emission^19,20,29–32^:

2 $${\rm{J}}={\rm{q}}\mu {N}_{c}E\exp [\frac{-q({\phi }_{T}-\sqrt{qE/\pi {\varepsilon }_{i}{\varepsilon }_{0}})}{kT}],$$

where *J*, $\mu$, *N*_*c*_, *qϕ*_*T*_, *E*, *ε*_*i*_, *ε*_0_, *k*, and *T* are the current density, electron drift mobility, density state in the conduction band, energy level of trap, electric field, dynamic permittivity, vacuum permittivity, Boltzmann’s constant, and absolute temperature, respectively. A trap energy *qϕ*_*T*_ of 1.1 eV was determined according to the Arrhenius plots presented in Fig. 3(a) at a temperature range from 298 to 398 K. An *ε*_*i*_ value of 4.3 was obtained from the slope of P–F plots and is lower than the static permittivity (*ε* = 7)^33^. P–F emission occurs due to electrons hopping between nearby traps^34^ which leads to a high HRS current, as shown in Fig. 1(b). Figure 3(b) displays the LRS *I–V* characteristics of the PECVD-SiN_x_ device that adheres to the SCLC under the most-negative voltage bias region.

**Figure 4:**
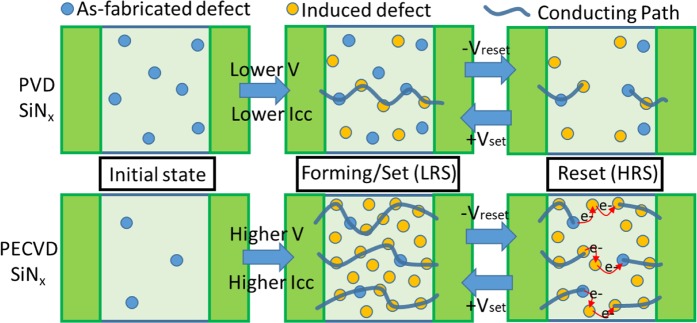
Schematic diagram of potential resistance switching in SiN_x_ RRAM.

Figure 4 illustrates the potential switching behaviours of the SiN_x_ RRAM device. The PVD-SiN_x_ dielectric has abundant defects in the initial condition because of its room temperature deposition with a low annealing temperature of 200 °C. The current conducting path can be formed and switched to LRS with a relatively low *I*_*cc*_ value and voltage. By contrast, the PECVD-SiN_x_ dielectric was deposited at a relatively high temperature of 300 °C, with fewer defects, so the conducting path could not be formed under low *I*_*cc*_ and voltage values. Therefore, a high forming voltage of 12 V and a high *I*_*cc*_ of 1 mA were required. Under such a high electric field, a large number of Si–N bonds were broken and excessive defects were created. The excessive defects in the PECVD-SiN_x_ layer may create multiple conduction paths and lead to a high HRS current, as shown in Fig. 1(b). To switch from LRS back to HRS, a negative *V*_*reset*_ was applied to rupture the conducting path. Although the conducting paths were ruptured, the electrons in the PECVD-SiN_x_ layer had a high possibility to conduct current in parallel ways via hopping through nearby defects. This further led to the high measured HRS current. Conducting paths can be formed again for electron transportation when a positive *V*_*set*_ is applied to the top electrode.

**Figure 5:**
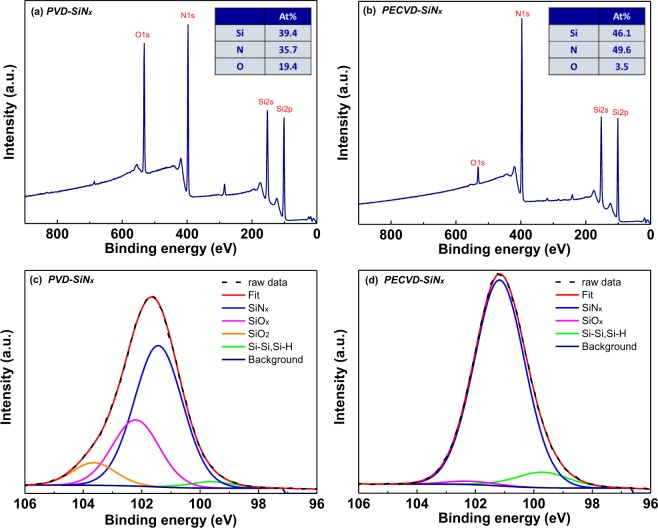
XPS data of (**a**) PVD-SiN_x_ (**b**) PECVD-SiN_x_ layers, and the related Si 2p spectrum of (**c**) PVD-SiN_x_ film (**d**) PECVD-SiN_x_ layers.

The deposited SiN_x_ layers were further analysed using X-ray photoelectron spectroscopy (XPS). Figure 5(a,b) depict the XPS data for PVD-SiN_x_ and PECVD-SiN_x_ layers, respectively. The related Si 2p spectra obtained from the XPS data are displayed in Fig. 5(c,d) for the PVD-SiN_x_ and PECVD-SiN_x_ layers, respectively. The N–Si composition of the as-fabricated PVD-SiN_x_ and PECVD-SiN_x_ layers were 0.91 and 1.08, respectively, which indicates a high number of defects inside the PVD-SiN_x_ layer. The nitride vacancies^21,35^ and Si dangling bonds^22,23^ can play crucial roles to form the conducting path. The slightly higher oxygen content in the PVD-SiN_x_ layer than in the PECVD-SiN_x_ layer cannot explain the significantly better device performance because the bond enthalpy of SO is higher than that of SN. That is, it is more difficult to break the SO bond to form the defects. Therefore, the XPS results support the proposed model depicted in Fig. 4 and adequately explain the measured *I–V* characteristics in Fig. 1 and the analysed current conduction mechanisms in Figs. 2 and 3. Such defect-assisted conduction is also the major mechanism for similar GeO_x_ RRAM devices^10^. The conducting path can be constructed or broken by the different polarities of the applied voltage.

**Figure 6:**
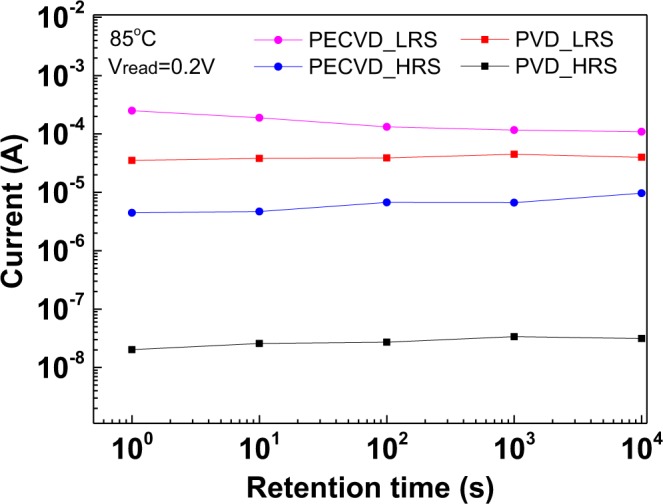
Retention test of N^+^-Si/PVD-SiN_x_/P^+^-Si and N^+^-Si/PECVD-SiN_x_/P^+^-Si RRAM devices under 85 °C.

**Figure 7:**
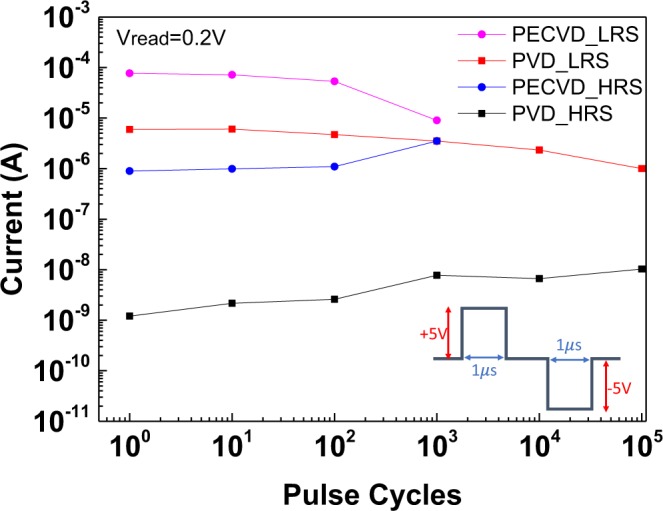
Endurance characteristics of N^+^-Si/SiN_x_/P^+^-Si RRAM devices.

Device endurance and data retention are the crucial characteristics of a nonvolatile memory. Figure 6 shows the retention data of PVD-SiN_x_ and PECVD-SiN_x_ RRAM devices under an 85 °C test condition. The PECVD-SiN_x_ device exhibits a much smaller resistance window and a rapid degradation from an initial value of 56 to 11 after 10^4^ s retention at 85 °C. The poor retention can be ascribed to the high forming voltage with large kinetic energy; then the electrons at 85 °C can partially annihilate or create a leakage path with multiple defects, as shown in Fig. 4. By contrast, the resistance window of an all-nonmetal PVD-SiN_x_ RRAM device slightly decreases from 1.8 × 10^3^ to 1.3 × 10^3^ after a retention time of 10^4^ s at 85 °C. The RRAM device endurance data are shown in Fig. 7, where the *V*_*set*_ and *V*_*reset*_ pulses of +5 V and −5 V were applied to devices with a 1-μs pulse width. The resistance window of the PECVD-SiN_x_ device degraded quickly, and the device failed after 10^3^ endurance cycles. Conversely, a large resistance window of two orders of magnitude was obtained for the PVD-SiN_x_ RRAM device, even after 10^5^ pulse cycles. The excellent endurance of the PVD-SiN_x_ device is related to the high number of defects that can be set and reset easily, with less destruction to the dielectric material.

**Figure 8:**
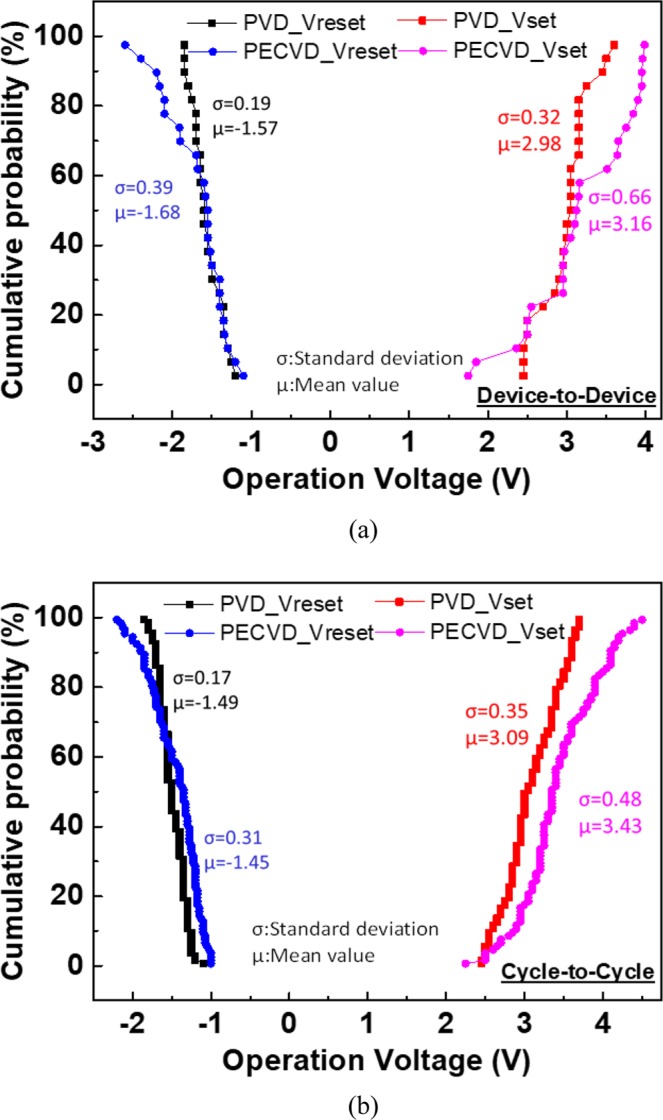
Operation voltage distributions of SiN_x_ RRAM devices for (**a**) device to device and (**b**) cycle-to-cycle.

Figure 8(a,b) display the device-to-device and cycle-to-cycle distributions of *V*_*set*_ and *V*_*reset*_ respectively, which are critical for the RRAM cross-point array^24–26^. The coefficient of variation (CV) was used to study the distribution and is defined as follows:

3 $${\rm{CV}}=\frac{\sigma }{|\mu |}\times 100 \% \,,$$

where *σ* is the standard deviation, and *μ* is the mean value. The lower CV value represents a tighter distribution and is crucial for a larger array size^26^. The device-to-device distributions were extracted from 25 different devices. The CVs of *V*_*set*_ and *V*_*reset*_ of the PVD-SiN_x_ RRAM were 10.7% and 12.1%, respectively, which are remarkably tighter than the 18.3% and 23.2% of PECVD-SiN_x_ devices, respectively. The cycle-to-cycle variations of the first 100 consecutive DC switching cycles, depicted in Fig. 8(b), also show the crucially tighter operation voltage distributions of PVD-SiN_x_ RRAM devices than those of PECVD-SiN_x_ ones. During the forming step, the dielectric soft breakdown must occur by breaking part of the SiN bonds. Based on the preceding discussion, the PECVD-SiN_x_ RRAM device requires a higher *I*_*cc*_ value and voltage during fabrication to create a conducting path that has an excessive number of defects and an uncontrollable reset current. These results further lead to poor *V*_*set*_ and *V*_*reset*_ distribution and fewer endurance cycles. One issue of nonmetal RRAM device is the relatively larger operation voltage than that of metal-oxide RRAM device, even though the operation voltage is still much less than that of a flash memory^36^. One possibility to lower the operation voltage is to use the weak bond-enthalpy GeO_x_ dielectric^9^, in which the defect-related conduction path can be formed by breaking the dielectric at low energy.

## Conclusion

High-performance all-nonmetal N^+^–Si/SiN_x_/P^+^–Si RRAM devices were achieved. The device performance was highly dependent on the deposition process. In this process, the current conduction in the RRAM device was highly related to the defects inside the SiN_x_ layer. The PVD-SiN_x_ RRAM devices exhibited favourable memory characteristics: a large memory window, high pulsed endurance, a long data retention time at 85 °C, and tight device-to-device *V*_*set*_ and *V*_*reset*_ distributions. The PVD-SiN_x_ RRAM device has high potential to realise a large-size cross-point memory array and to be embedded in CMOS technology to realise electronic neurons.

## Methods

A P^+^ silicon wafer was used as a bottom gate. After conducting the RCA clean for the wafer and dipping the wafer into dilute HF to remove the native oxide, a 25-nm-thick SiN_x_ layer was formed by either PVD using electron-beam evaporation or PECVD. Additional furnace annealing was applied to the PVD-SiN_x_ layer at 200 °C in a N_2_ ambient for 30 min. For the PECVD-SiN_x_ layer, a NH_3_ of 6 sccm, 8% SiH_4_ in Ar of 125 sccm, and N_2_ of 200 sccm were used at a temperature of 300 °C. Finally, the N^+^–Si top electrode was fabricated as the top electrode with a diameter of 120 μm^11^. The electrical characteristics were measured using the HP4155B analyser. The pulse stress was generated using a pulse generator (81110, Agilent). Material analysis was performed through XPS analysis by using Thermo Scientific K-Alpha with an X-ray spot size of 400 μm.
